# Resistance training effects on pubertal children with a risk of developing pediatric dynapenia

**DOI:** 10.1007/s00421-024-05436-z

**Published:** 2024-02-29

**Authors:** Cassio V. Ruas, Sébastien Ratel, Kazunori Nosaka, Gabriela Castellano, Ronei S. Pinto

**Affiliations:** 1 School of Medical and Health Sciences, Edith Cowan University, 270 Joondalup Drive, Joondalup, WA 6027 Australia; 2 Brazilian Institute of Neuroscience and Neurotechnology-Institute of Physics Gleb Wataghin, Universidade Estadual de Campinas, R. Sérgio Buarque de Holanda, 777, Campinas, São Paulo, 13083-859 Brazil; 3 Université Clermont Auvergne, AME2P, 63000 Clermont-Ferrand, France; 4 Exercise Research Laboratory, School of Physical Education, Physiotherapy and Dance, Universidade Federal Rio Grande do Sul, Porto Alegre, Brazil

**Keywords:** Strength training, Maximal voluntary contraction, Muscle quality, Echo-intensity, Muscle thickness, Body composition

## Abstract

**Purpose:**

Many modern-day children are at risk of pediatric dynapenia (muscle weakness). We examined the effects of a 12-week resistance training (RT) program on neuromuscular function and body composition parameters in pubertal children with a risk of dynapenia.

**Methods:**

Twelve children (13.4 ± 0.9 y) with dynapenia performed a progressive RT program consisting of knee extension and flexion, bench press, abdominal crunch, back extension, lateral pull-down, elbow flexion, and upright row (1–2 sets of 10–15 repetitions/exercise) twice/week for 12 weeks. Outcome measures included one-repetition maximum (1-RM) strength, maximal voluntary isometric contraction (MVIC) torque, rate of torque development (RTD), electromyographic (EMG) activity, muscle thickness (MT), muscle quality (MQ) assessed by echo intensity (MQ_EI_) of the knee extensors and specific tension of MVIC torque to thigh fat-free mass (MQ_ST_), and total and regional body and bone composition assessed by dual-energy X-ray absorptiometry. Changes in the measures before and after the 12-week RT and associations among the measures were analyzed by linear mixed models.

**Results:**

Significant (*p* < 0.05) increases in 1-RM (63.9 ± 4.5%), MVIC torque (16.3 ± 17.8%), MT (18.8 ± 5.5%) and MQ (MQ_EI_: -25.9 ± 15.2%; MQ_ST_: 15.1 ± 18.8%;) were evident from pre- to post-training. Total fat-free mass (FFM) increased by 2.3 ± 3.2% from baseline (*p* = 0.01), but no changes (*p* > 0.05) in the other measures were observed. Significant (*p* < 0.05) associations between the changes in 1-RM and/or MVIC torque and the changes in quadriceps MT, MQ_EI_, MQ_ST_ and total body FFM were evident.

**Conclusions:**

The 12-week RT was effective for improving neuromuscular and body composition parameters, and thereby reversed the risk of pediatric dynapenia.

## Introduction

Children and adolescents have become less physically active over the last decades. For example, epidemiological studies have shown that only 24% of children and adolescents (i.e., 6–17 years old) perform 60 min of moderate to vigorous daily physical activity in the United States (Katzmarzyk et al. 2018). A similar decline in physical activity levels has also been reported in other countries such as Australia, Ethiopia, Mexico, Portugal and Brazil (Aubert et al. 2018). A study in the UK reported that 7-year-old English children spent 50% of their waking day time sedentary, and that this sedentary time increased up to 75% at the age of 15 years old (Janssen et al. 2016). It should be noted that children and adolescents appear to have more sedentary time than adults (Matthews et al. 2008; Colley et al. 2011). The increased sedentarism among children and adolescents seems to be partly attributed to a significant reduction of school physical education activities, and to the technological advances, which have decreased children’s exposure to “free play” and their need to move (Faigenbaum et al. 2019).

Other factors that could increase sedentary behavior of children and adolescents are the lack of family members (e.g., guardians, parents) who continuously encourage them to perform physical activities and sports (e.g., daily physical education classes, outdoor time), reduced access of children to school facilities (e.g., gymnasium, sporting field, outdoor playgrounds), living in communities/environments that do not have infrastructures (e.g., well-maintained facilities, playgrounds and parks) that are safe to use and facilitate physical activity for youth, and the lack of physical promotion strategies and initiatives for youth (Katzmarzyk et al. 2018; Aubert et al. 2018). Thus, the decreased level of physical activity in modern-day children and adolescents is concerning, since physical inactivity in early life is known to lead to sedentary behavior during adulthood (Laurson et al. 2012).

Physical inactivity levels in children have recently been increased more by the COVID-19 pandemic (Shuffrey et al. 2022; Moyer 2022). It has been reported that infants who were born during the pandemic period have lower scores on tests of gross and fine motor skills when compared with those born before the pandemic (Shuffrey et al. 2022). This seems to be related to the limited playtime and social interactions of children, reducing even more their physical activity levels during the COVID-19 pandemic (Moyer 2022). Post-pandemic reports also revealed that primary school-aged children have reduced outdoor play or exercise time in their daily routines over the years after the lockdown period, leading to increased adiposity (Sum et al. 2022).

Longitudinal data have shown that modern-day children and adolescents are weaker and less physically active than previous generations (Laurson et al. 2012; Cohen et al. 2011), and that the sedentary behavior of contemporary youth has resulted in dynapenia (loss of muscle strength), which is a condition that was previously seen in only older adults (Faigenbaum et al. 2018a, 2019; Faigenbaum and MacDonald 2017). The term pediatric dynapenia is characterized by low levels of muscular strength and power and consequent functional limitations not caused by neurologic or muscular disease in children (Faigenbaum et al. 2019; Faigenbaum and MacDonald 2017). This is alarming because pediatric dynapenia can lead to consequent functional limitations and co-morbidities along the lifespan, and a reduced strength production capacity is associated with an increase in obesity (Faigenbaum et al. 2019).

A recent meta-analysis including only prospective cohort studies with a follow-up period of at least 1 year found that youth with higher levels of muscular fitness tested by handgrip, sit-ups, long jump, among others presented lower adiposity and cardiometabolic complications along with greater bone health later in life (García-Hermoso et al. 2019). Furthermore, Henriksson et al. (2019) in a prospective cohort study of 1.2 million participants showed that increased handgrip and knee extension muscle weakness during adolescence were strongly associated with disabilities 30 years later in life. Other prospective studies have also shown that youth with muscle weakness have increased risk of cardiovascular disease and diabetes during midlife (Fraser et al. 2021; Timpka et al. 2014). Thus, appropriate resistance training (RT) interventions during childhood that prevent strength loss are necessary to reduce or prevent the risk of developing pediatric dynapenia (Faigenbaum et al. 2019, 2023; Faigenbaum and MacDonald 2017). Strengthening exercises are also essential for the neuromuscular, cognitive and cortex structural development of youth, which influence on neurocognitive and motor development in later stages of life (Myer et al. 2015).

Several review articles have shown that structured RT programs are effective for increasing neuromuscular function such as muscle strength and body composition variables, and/or avoid health complications in children (Faigenbaum et al. 2018a, 2019; Faigenbaum and MacDonald 2017). However, the effects of RT programs in children with a risk of developing pediatric dynapenia have not been systematically investigated. The present study examined changes in muscle strength and other neuromuscular parameters, together with body composition following a RT program in sedentary children. In particular, an important variable to assess in this context is muscle quality (MQ), which may indicate the amount of the contractile components of the muscle and non-contractile tissue (e.g., intramuscular fat infiltration) (Radaelli et al. 2013) and/or the ability to generate force relative to segmental muscle “size” or “lean mass” (Goodpaster et al. 2006; Mota et al. 2017), since intramuscular lipid infiltration in the skeletal muscle may result in insulin resistance and associated complications in youth (Weiss et al. 2005). However, MQ has not been extensively explored in studies of children, in particular using echo-intensity (EI) to estimate intramuscular fat infiltration, as previously investigated in adults and older adults (Henriksson et al. 2019; Mota et al. 2017).

To the best of our knowledge no previous study has systematically investigated effective resistance training programs for reduction of pediatric dynapenia. It may be possible to do that by examining whether linear associations exist between changes in muscle strength with changes in body composition and neuromuscular parameters following a RT program for children who have reduced levels of muscular strength. Therefore, the aim of the present study was to investigate the effects of a 12-week RT program on neuromuscular and body composition parameters in pubertal children with a risk of dynapenia. It was hypothesized that the 12-week of RT would increase one repetition maximal (1-RM) strength, maximal voluntary isometric contraction (MVIC) torque, rate of torque development (RTD), electromyographic (EMG) activity, muscle thickness (MT), and muscle quality (MQ) measured by both echo intensity (MQ_EI_) and specific tension of MVIC torque per unit of thigh fat-free mass (FFM) of the knee extensors (MQ_ST_), and reduce body fat percentage, but provide no changes in bone mineral density (BMD) and bone mineral content (BMC) because of the limited duration of training. We also hypothesized that there would be linear associations between changes in muscle strength, neuromuscular parameters and body composition.

## Methods

### Participants

Twelve pubertal children (7 boys and 5 girls) from southern Brazil who had not performed any RT and had never been engaged in any other structured exercise/training program participated in the study. All of them were free from any neuromuscular injury. This recruitment criterion was used as it was unlikely that resistance trained children would present concerning levels of muscle weakness and/or an increased risk of developing pediatric dynapenia (Faigenbaum et al. 2019, 2023; Faigenbaum and MacDonald 2017). Their baseline average (range) age, standing height, body mass, body mass index (BMI) and Tanner stage were 13.4 ± 0.9 (12–15) y, 163.4 ± 7.8 (154.9–177.5) cm, 53.0 ± 7.8 (39.4–61.8) kg, 13.1 ± 2.5 (8.8–15.9) kg.m^2^, and 3.4 ± 0.6 (2.5–4.5), respectively. The average values of the above parameters were 13.4 ± 1.0 (12–15) y, 167.2 ± 8.3 (154.9–177.5) cm, 55.0 ± 7.6 (39.4–59.8) kg, 14.5 ± 1.8 (10.7–15.9) kg.m^2^ and 3.6 ± 0.5 (3.0–4.5) for the boys, and 13.4 ± 1.14 (12–15) y, 158.1 ± 2.0 (155.4–160.8) cm, 50.2 ± 8.1 (40.4–61.8) kg, 11.0 ± 1.7 (8.8–13.2) kg.m^2^ and 3.0 ± 0.6 (2.5–3.5) for the girls. Their baseline knee extensor MVIC torque normalized by body mass (MVIC/BM) was 2.68 ± 0.48 N m kg^–1^ in boys, and 2.34 ± 0.48 N m  kg^–1^ in girls. These were in a similar level to or lower than those of severely obese untrained male and female adolescents, and also lower than most physically active non-obese youth of the same age group (12–15 years) reported in the last two decades (Table 1). The baseline bilateral leg extension strength normalized by body mass [1-RM (kg)/BM (kg)] of the children in the present study (boys: 0.62 ± 0.13; girls: 0.46 ± 0.06) was also ~ 67% lower than that of 14-year-old physically active male adolescents reported by a previous study (Pullinen et al. 2011). These indicate that the participants of the present study were at risk of developing pediatric dynapenia.

**Table 1:** Baseline knee extensor maximal voluntary isometric contraction torque of the knee extensors (MVIC) normalized by body mass (MVIC/BM) reported by previous studies in children with a similar age range to the sample of the present study (12–15 years)

Research study	Study year	Age (y)	Status	Sex	Equipment	Knee joint angle	MVIC/BM (N m kg^−1^)
Maffiuletti et al. (2008)	2008	14.9 ± 1.1	Nob	B	CYBEX	80°	3.6
Abdelmoula et al. (2012)	2012	14.4 ± 0.7	Nob	B	Home-made	60°	3.3
Abdelmoula et al. (2012)	2012	14.2 ± 1.4	Ob	B	Home-made	60°	2.5
Garcia-Vicencio et al. (2015)	2015	13.6 ± 0.8	Nob	G	CYBEX	80°	3.2
Garcia-Vicencio et al. (2015)	2015	13.9 ± 0.9	Ob	G	CYBEX	80°	2.8
Gillen et al. (2020)	2020	13.9 ± 0.8	Nob	B	BIODEX	60°	2.2

*Ob* obese; *Nob* non obese; *G* girls; *B* boys

Based on the data from a previous study reporting changes in leg extension 1-RM from before (19.3 ± 9.0 kg) to after (27.2 ± 10.9 kg) an 8-week moderate-load RT program performed by 16 untrained pubertal children (Faigenbaum et al. 1999), the effect size was estimated to be 0.9. Using G*Power 3.1 (Institute for Experimental Psychology, Dusseldorf, Germany), with a power of 0.8 and a significance level of 0.05, the required sample size was estimated to be 10. Accounting for possible dropouts and an estimation error, a total of 14 participants were recruited initially, but two children did not attend the first session and thus did not continue the training for 12 weeks. Thus, the analyses were based on the 12 participants who completed all the 24 training sessions.

The children and their parents/guardians were informed about the risks and benefits of participating in the present study, and all of them provided a written informed consent. They also completed a pre-exercise medical questionnaire to reduce any health or accident risks upon participation in the present study. All the procedures of the study were approved by the University’s Research Ethics Committee (No 1.802.716) and were in accordance with the standards of the Declaration of Helsinki.

### Experimental design

The participants performed a full body RT twice a week for 12 weeks as detailed below. However, measurements were focused on knee extensor muscles, because these muscles are commonly used in children’s functional tasks and activities. The outcome measures of neuromuscular and body composition variables were taken 3–5 days before the first training session (pre) and 3–5 days after (post) the last training session. Pre- and post-testing consisted of two sessions separated by at least 24 h. On the first session, participants had the following assessments in the order: (1) anthropometric (standing height, body mass, Tanner stage) measures, (2) ultrasound (MT and EI) and DXA-derived body composition measures (body fat percentage, BMD, BMC, total body and thigh FFM, thigh fat percentage and fat mass) and (3) MVIC torque and RTD of the knee extension, and EMG activity of the rectus femoris (RF) and vastus lateralis (VL). On the second session, participants were measured for 1-RM. The ultrasound and isokinetic measurements were always taken from the right leg while the 1-RM was performed bilaterally by the participants. In addition, DXA scans were taken from both full body and the right thigh specific region. Outcome measures were compared between before and after the 12 weeks of RT, and the associations between the significant changes among variables were tested.

### Resistance training and 1-RM strength

The RT program was performed two sessions a week (60–90 min a session) for 12 weeks, with each session separated by at least 48 h (total of 24 sessions). In each session, participants performed the following exercises: bilateral knee extension, bilateral knee flexion, bench press, abdominal crunch, back extension, lateral pull-down, elbow flexion and upright row. The exercises were performed using weight stack and cable machines (Konnen Gym, China). Before each training session, participants performed a 5-min warm-up on a cycle ergometer (Movement Technology, BM 2700, Movement, Pompeia—SP, Brazil).

Volume and intensity of training were increased in a linear manner as shown in Table 2. The volume varied from 1 set of 15 repetitions to 2 sets of 10 repetitions, while intensity increased from 55 to 80% of 1-RM from weeks 1 to 12. For every exercise, concentric and eccentric contractions were performed by lifting and lowering the load under control for ~ 4 s (~ 2 s for each contraction type) through a full range of motion. Exercises were performed in a constant flow with no rest (pause) between repetitions and/or contractions. Two minutes of rest were given between each set. All participants were familiarized with the training protocol at the beginning of the first training session, and the sessions were systematically supervised by a trained instructor.

**Table 2:** Resistance training program. Number of sessions, sets, repetitions and intensity over 12 weeks

Week	Number of sessions	Number of sets	Number of repetitions	Intensity (% of 1-RM)
1	2	1	15	55–60%
2	2	2	15	55–60%
3	2	2	15	55–60%
4	2	2	15	55–60%
5	2	2	12	60–65%
6	2	2	12	60–65%
7	2	2	12	65–70%
8	2	2	12	65–70%
9	2	2	10	70–75%
10	2	2	10	70–75%
11	2	2	10	75–80%
12	2	2	10	75–80%

To determine the training intensity, the 1-RM of each exercise was tested before the training session. Participants first warmed up by performing 8–10 repetitions with a light load (i.e., 20% of participants’ body mass). The load was then increased until the participants failed to perform the repetition through the full range of motion during ~ 4 s. The lifting and lowering times were controlled by the use of a metronome. A maximum of four attempts was given for the 1-RM to be reached, with 4-min rest between attempts. The 1-RM was defined as the heaviest load that each participant could complete a single repetition for each exercise. The 1-RM was also tested after 6 weeks of training to adjust the training load. Although this was done for all exercises, only the bilateral knee extensor 1-RM recorded before and after 12 weeks of training were used for further analyses.

### Outcome measures

#### Anthropometric measures

Participants were measured barefoot for standing height on a wall-mounted stadiometer (Seca 213 stadiometers, Germany) and body mass on a digital scale (Filizola scale, Brazil, accuracy 0.1 kg). Body mass index was calculated by body mass (kg) divided by standing height^2^ (m^2^). In addition, biological maturity was assessed by parents or guardians from pictures based on pubic hair and testicular/penis development for boys and pubic hair and breast development for girls (Tanner 1962).

#### Ultrasound measures

Each participant lay supine on a table with legs relaxed for 10 min to allow body fluids to stabilize. The investigator measured MT and EI of the vastus lateralis (VL), vastus medialis (VM), rectus femoris (RF) and vastus intermedius (VI) of the right lower limb using a real-time B-mode ultrasound apparatus (Nemio XG SSA-580 A, Thoshiba., Japan) with a linear array probe (38 mm, frequency band 9.0 MHz). Measurements were performed after applying a water-soluble gel between skin and transducer to provide acoustic contact. The probe was placed at 50% of the distance between the lateral condyle and greater trochanter of the femur for the measurements of the VL muscle, 60% of this distance for the measurements of the RF muscle, and 30% of this distance for the measurements of the VM muscle (Ruas et al. 2018).

The largest distance between the adipose muscle upper fascia and the lower fascia was determined for all muscles except for VI, which was measured at the distance between the bone and the adipose-muscle upper fascia (Ruas et al. 2017, 2018). The straight-line function of the ImageJ software (Version 1.48v, National Institutes of Health, Bathesda, MD, USA) was used for MT analyses. The average of three reproducible measurements (< 5% difference) was considered as the MT for each muscle. Quadriceps MT was then calculated as the sum of MT of RF, VI, VL and VM (Ruas et al. 2019).

EI values were obtained in the greatest region of interest of each quadriceps head by surrounding the muscles without including fascia or bone, and then using the histogram function of the ImageJ software. EI values were expressed in a range between 0 (black) and 255 (white) (Ruas et al. 2017). The average of three reproducible EI measurements (< 5% difference) was calculated for each muscle of the quadriceps, and quadriceps EI was considered as the total EI of RF, VI, VL and VM. As indicators of muscle quality (MQ), two parameters were used: (1) MQ_EI_ was derived from EI measurements, in which a decrease in MQ_EI_ from pre- to post-training would represent an increase in MQ (Ruas et al. 2017); (2) MQ_ST_ was calculated by dividing MVIC torque by thigh FFM or “lean mass”, in which an increase in MQ_ST_ (i.e., “specific tension” of MVIC) from pre- to post-training would represent an increase in MQ (Goodpaster et al. 2006; Mota et al. 2017).

For all analyses, the transducer was placed perpendicular to the muscle fibers. The same ultrasound settings for gain (90 dB) and image depth (70 mm) were used for all ultrasound assessments to optimize the quality of the images and allow consistent measurements across participants (Ruas et al. 2017). An experienced researcher in ultrasound measurements performed the assessments.

#### Body composition measures

**Fig. 1:**
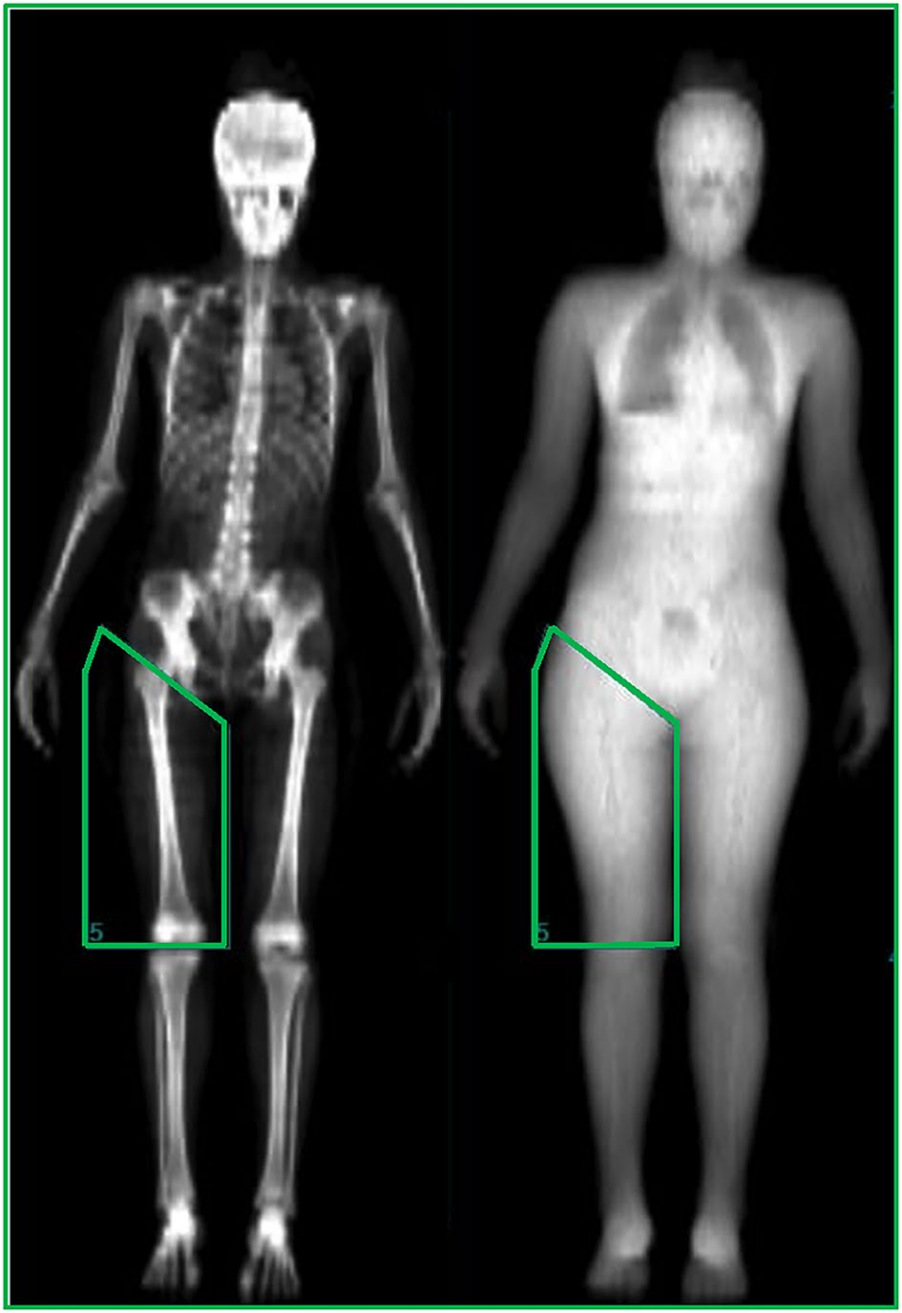
Dual-energy X-ray absorptiometry (DXA) image showing a full body X-ray scan from a single representative participant. Green traces show the thigh-specific measures that were performed using straight line borders (from the head of the femur to the tibial edge) to form a “thigh box” as a region of interest

Body composition was assessed using DXA (Hologic Discovery W, EUA). Participants lay supine on a table and were asked to remain relaxed with their hips and shoulders aligned and centralized, and feet rotated medially for a full body X-ray scan from head to toe direction to be taken for approximately 7 min. The full body fat percentage, fat mass, FFM, BMD and BMC were further measured and recorded from the scan. Since most of the neuromuscular variables included in the present study were specific to thigh muscle groups (i.e., quadriceps), specific measures of the right thigh region (thigh-specific fat percentage, fat mass and FFM) were also performed and recorded for further analyses. For this, the right thigh region of the scan was demarcated during offline analyses using straight line borders from the head of the femur to the tibial edge to form a “thigh box” as a region of interest (see Fig. 1). It is worth noting that the radiations emitted from the DXA assessments are less than 1 µSv per session, which do not represent health risks in humans, and thus have been previously used to measure body composition in children (dos Cunha et al. 2015). The equipment was also calibrated daily during the experiment and before each assessment according to the guidelines of the DXA company model.

#### Maximal voluntary isometric contraction (MVIC) torque

Participants were tested for MVIC torque of the right knee extensors on an isokinetic dynamometer (Cybex Norm, USA). Torque signals were recorded, digitized and sampled at 2 kHz using Miograph software with a 14 bit analogue-to-digital converter (New Miotool System, Miotec, Brazil, RS), and further analyzed offline using a custom-made routine developed in MATLAB (Version 6.5, Mathworks, USA).

All participants sat upright on the dynamometer, and had straps across their thighs, chest and hips to minimize movements from other parts of the body during the test. The tested lower limb of the participants was attached to the lever arm of the dynamometer two centimeters above the medial malleolus, and the axis of rotation of the dynamometer was aligned to the femur’s lateral epicondyle. Before testing, participants warmed up by performing light (submaximal) isokinetic knee extension and flexion contractions at 120°s^−1^. Then, participants performed MVIC, which consisted of three 5-s knee extension maximum attempts with the lower limb fixed at 60° of knee flexion (0° = full knee extension), with 2-min rest between trials. Strong verbal encouragement was given to each participant to extend the knee as strong and fast as possible, without performing any countermovement or pretension before the trials. Real-time torque feedback was displayed on a screen in front of participants. The highest value of the three MVIC peak torques was used for further analysis.

#### Rate of torque development (RTD)

RTD was calculated as the average slope of the MVIC torque-time curve at intervals 0–50, 0–100, 0–150, 0–200, 0–250, 0–300 and 0–350 ms. The onset of muscle contraction was defined as 2.5% of each individual MVIC torque. The MVIC with the highest peak torque of the three trials for each participant was used for calculating the RTD values for each time interval.

#### Electromyographic (EMG) activity

During MVIC testing, EMG activity from the RF and VL of the right lower limb were recorded by a Miotool EMG system (New Miotool System, Miotec, Brazil, RS) using two bipolar surface electrodes (Ag–AgCl) positioned in the longitudinal direction of each muscle aligned to the muscle fibers. The anatomical sites used for placement of the electrodes for each muscle were selected according to the “Surface EMG for Non-Invasive Assessment of Muscles (SENIAM) guidelines” (Hermens et al. 1999). A ground electrode was placed over the tibial tuberosity of the lower limb tested. The skin was shaved, abraded and cleaned with 70% isopropyl alcohol swabs. Raw EMG signals were filtered at 20–500 Hz (band pass filter), amplified (1000x) and digitized at 2 kHz. Root mean square (RMS) of EMG signals was calculated over the period of 1 s around the time of peak torque during the MVIC trials. The EMG values (RMS) of the MVIC with the highest peak torque were then normalized to peak torque (EMG/torque). All data for EMG activity were analyzed using Miograph software (14 bit analogue-to-digital converter, Miotec, Brazil).

### Statistical analyses

Data were screened for normality of distribution and homogeneity of variances using the Shapiro–Wilk test and the Levene’s test, respectively. The dependent variables (MVIC torque, RTD, EMG, MT, MQ_EI_, MQ_ST_, body fat percentage, BMD, BMC, total body and thigh FFM, thigh fat percentage, thigh fat mass) measured before and after RT were then compared using a series of linear mixed models with a repeated measures design, with time (pre- to post-training) used as a fixed effect. The use of linear mixed models with a repeated measures design has been previously recommended for experimental studies examining changes in variables from before to after training interventions, as this type of statistical analysis allows grouping of individuals and/or variables from different units of measurement, reducing inter-individual variability and eliminating any confounding variables (Moura et al. 2021). These models also allow describing the relationship between a response variable and other explanatory variables obtained along with this response variable, in which at least one of the explanatory variables is a categorical grouping variable representing an experimental “unit” (Magezi 2015).

The most appropriate covariance structure of each model was first determined by visually inspecting the variances using descriptive statistics, and then testing the correlations between the time points of each variable by Pearson correlation tests (*r*). These analyses indicated that “compound symmetry”, “unstructured” and “scaled identity” types of covariance structures should be used in the models. Additional linear mixed model analyses were also conducted to test whether linear associations existed between the significant changes in muscle strength and the changes in the neuromuscular and body composition variables from pre- to post-training. For these models, “time” (pre- to post-training) was used as a fixed effect and “participants” was used as a random effect, while MVIC torque and 1-RM were used as covariates, and the neuromuscular and body composition parameters were used as dependent variables. Significance was set at *p* < 0.05. All analyses were performed with SPSS 21.0 (Statistical Package for Social Sciences, Armonk, NY, USA).

## Results

### Training

All children completed the entire training program (24 sessions in 12 weeks) and appeared to enjoy the program.

### MVIC torque, 1-RM, RTD and EMG

**Fig. 2:**
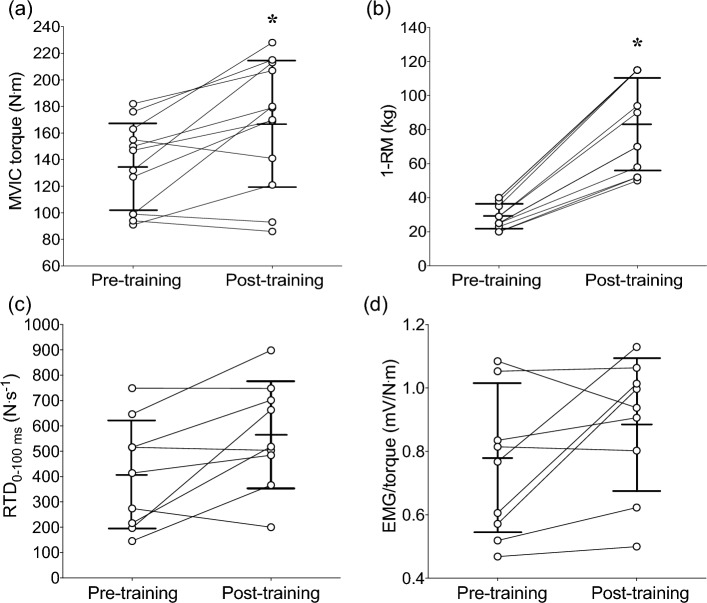
Changes in knee extensor maximal voluntary isometric contraction (MVIC) torque (**a**), one-repetition maximum (1-RM) **(b)**, rate of torque development (RTD) at 0–100 ms interval (**c**), and vastus lateralis muscle electromyographic (EMG) activity normalized to knee extensor MVIC torque (EMG/torque) (**d**) from pre- to post-training. Vertical lines indicate mean ± SD values of 12 participants, circles represent individuals, and horizontal lines indicate individual changes from pre- to post-training. *Indicates significant difference from pre-training (*p* < 0.05)

Linear mixed models revealed significant time effects for changes in MVIC torque [*t*(11) = −3.47, *p* = 0.005)] and 1-RM [*t*(11) = −9.19, *p* < 0.001)] from pre- to post-training. MVIC torque at baseline was 134.5 ± 32.6 N m (range: 99.0–176.0), and increased by 16.3 ± 17.8% (−8.5–45.0%) from pre- to post-training (Fig. 2a). 1-RM at baseline was 29.1 ± 7.4 kg (20–40 kg) and increased by 63.9 ± 4.5% (55.8–69.6%) from pre- to post-training (Fig. 2b). Similarly, when normalized to body mass (BM), MVIC torque (MVIC/BM) and 1-RM (1-RM/BM) significantly increased by 14.6 ± 17.9% [*t*(11) = −2.93, *p* = 0.014)] and 63.2 ± 4.6% [*t*(11) = −9.55, *p* < 0.001] from pre- to post-training, respectively. Since the normalization to BM did not seem to influence the results, absolute MVIC torque and 1-RM were considered for further statistical analyses and interpretation of the results.

In contrast, no significant time effects were observed for RTD at 0–50 [*t*(16) = −1.41, *p* = 0.18], 0–100 [*t*(16) = 1.33, *p* = 0.20] (Fig. 2c), 0–150 [*t*(16) = −1.64, *p* = 0.12], 0–200 [*t*(16) = −1.71, *p* = 0.11], 0–250 [*t*(16) = −1.82, *p* = 0.87], 0–300 [*t*(16) = −1.96, *p* = 0.68] and 0–350 ms [*t*(16) = −2.07, *p* = 0.55] intervals, and for RF EMG/torque [*t*(8) = −0.74, *p* = 0.48] (Table 3) and VL EMG/torque [*t*(8) = −2.01, *p* = 0.78] (Fig. 2d). Similarly, no significant time effects were found when RTD was normalized to BM (RTD/BM) in all time intervals (*p* > 0.53). It is worth noting that due to technical problems during data collection (e.g., unforeseen countermovement torque in the force–time curve, and clipping of the EMG recording) in the pre- or post-training measures, RTD and EMG data were analyzed for nine children.

**Table 3:** Changes (mean ± standard deviation and ranges of 12 participants) in the rate of torque development (RTD) over different time intervals (0–100, 0–150, 0–200, 0–250, 0–300, 0–350 ms), rectus femoris (RF) electromyographic activity (EMG) normalized to knee extensor maximal voluntary isometric contraction torque (EMG/torque), total body fat mass, thigh fat mass, thigh fat percentage, thigh fat free mass (FFM), and thigh bone height (distance from the greater trochanter to the medial line of the patella) from before (pre) to after training (post)

Variable		Pre	Post	*p*
RTD 0–50 ms (N s^−1^)	Mean	395.9	407.9	0.18
	± SD	± 224.8	± 213.4	
	Range	26.6–704.9	193.8–915.0	
RTD 0–100 ms (N s^−1^)	Mean	541.3	407.9	0.21
	± SD	± 213.4	± 213.4	
	Range	145.3–748.9	299.2–897.9	
RTD 0–150 ms (N s^−1^)	Mean	364.3	514.3	0.12
	± SD	± 204.2	± 184.3	
	Range	92.1–676.0	202.2–760.2	
RTD 0–200 ms (N s^−1^)	Mean	314.6	450.5	0.87
	± SD	± 182.5	± 152.4	
	Range	49.3–579.4	204.2–675.9	
RTD 0–250 ms (N s^−1^)	Mean	149.8	392.9	0.87
	± SD	± 156.6	± 127.3	
	Range	31.8–492.9	203.5–605.7	
RTD 0–300 ms (N s^−1^)	Mean	232.7	344.8	0.68
	± SD	± 134.2	± 107.6	
	Range	21.8–422.4	217.8–535.1	
RTD 0–350 ms (N s^−1^)	Mean	203.7	306.9	0.55
	± SD	± 116.9	± 92.9	
	Range	15.9–366.5	191.4–475.4	
RF EMG/torque (mV/N m)	Mean	0.9	1.0	0.36
	± SD	± 0.5	± 0.5	
	Range	0.3–2.0	0.06–1.6	
Total body fat mass (kg)	Mean	13.5	13.5	0.59
	± SD	± 4.9	± 5.1	
	Range	6.6–22.8	7.1–22.6	
Thigh fat mass (kg)	Mean	5.9	5.9	0.81
	± SD	± 1.8	± 1.7	
	Range	3.3–9.0	3.4–9.2	
Thigh fat percentage (%)	Mean	29.7	29.5	0.62
	± SD	± 7.3	± 7.1	
	Range	17.4–39.3	18.3–39.2	
Thigh FFM (kg)	Mean	13.1	13.3	0.26
	± SD	± 2.5	± 2.7	
	Range	8.8–15.8	9.3–16.8	
Thigh bone height (cm)	Mean	42.0	42.3	0.18
	± SD	± 2.3	± 2.7	
	Range	38.7–45.4	39.9–46.7	

### Muscle thickness and muscle quality

**Fig. 3:**
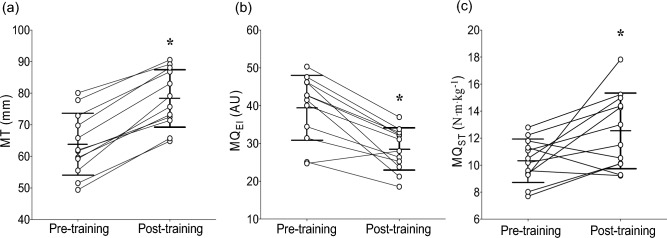
Changes in muscle thickness (MT, **a**) and muscle quality measured by echo intensity (MQ_EI_, **b**) and by the specific tension of knee extensor maximal voluntary isometric contraction torque per unit of thigh fat free mass (MQ_ST_, **c**) from pre- to post-training. Vertical lines indicate mean ± SD values of 12 participants, circles represent individuals, and horizontal lines indicate individual changes from pre- to post-training. *Indicates significant difference from pre-training (*p* < 0.05)

Significant time effects were found for MT [*t*(11) = −12.24, *p* < 0.001], MQ_EI_ [*t*(11) = 5.59, *p* < 0.001] and MQ_ST_ [t(22) = −2.38, *p* = 0.026] from pre- to post-training. MT increased by 18.8 ± 5.5% (range: 10.2–29.8%), MQ_EI_ decreased by −25.9 ± 15.2% (−48.8–11.7%), and MQ_ST_ increased by 15.1 ± 18.8% (−17.9–47.9%) from pre- to post-training (Fig. 3).

### Body composition

**Fig. 4:**
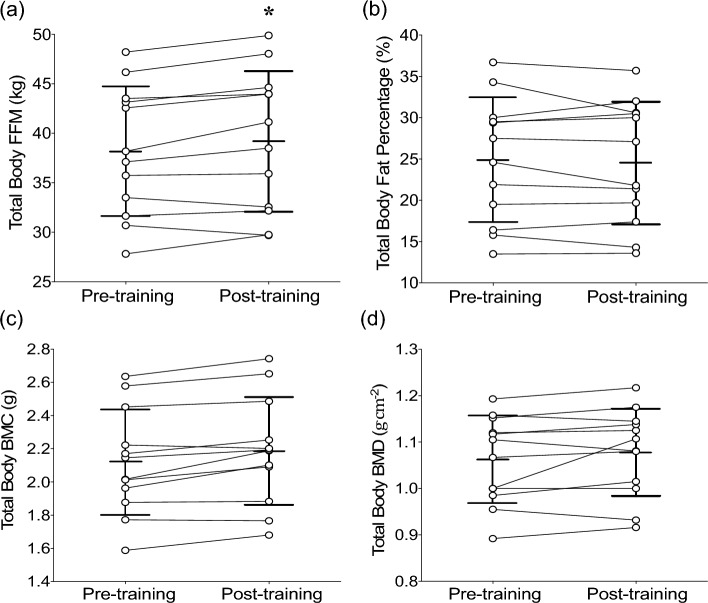
Changes in total body fat-free mass (FFM, **a**), body fat percentage (**b**), bone mineral content (BMC, **c)**, and bone mineral density (BMD,** d**) from pre- to post-training. Vertical lines indicate mean ± SD values of 12 participants, circles represent individuals, and horizontal lines indicate individual changes from pre- to post-training. *Indicates significant difference from pre-training (*p* < 0.05)

A significant time effect was found for total body fat-free mass (FFM) [*t*(11) = −2.89, *p* = 0.014] (Fig. 4a), which increased after training by 2.3 ± 3.2% (−3.4–7.2%) from baseline. However, no changes were evident for total body fat percentage [*t*(11) = 0.88, *p* = 0.39] (Fig. 4b), BMC [*t*(22) = −1.04, *p* = 0.31] (Fig. 4c), BMD [*t*(11) = −1.85, *p* = 0.091] (Fig. 4d), and total body [*t*(11) = 0.56, *p* = 0.59] and thigh fat mass [*t*(11) = −0.25, *p* = 0.81], thigh fat percentage [*t*(11) = 0.51, *p* = 0.62] and thigh FFM [*t*(11) = −1.18, *p* = 0.26] (Table 3).

### Associations between variables

**Fig. 5:**
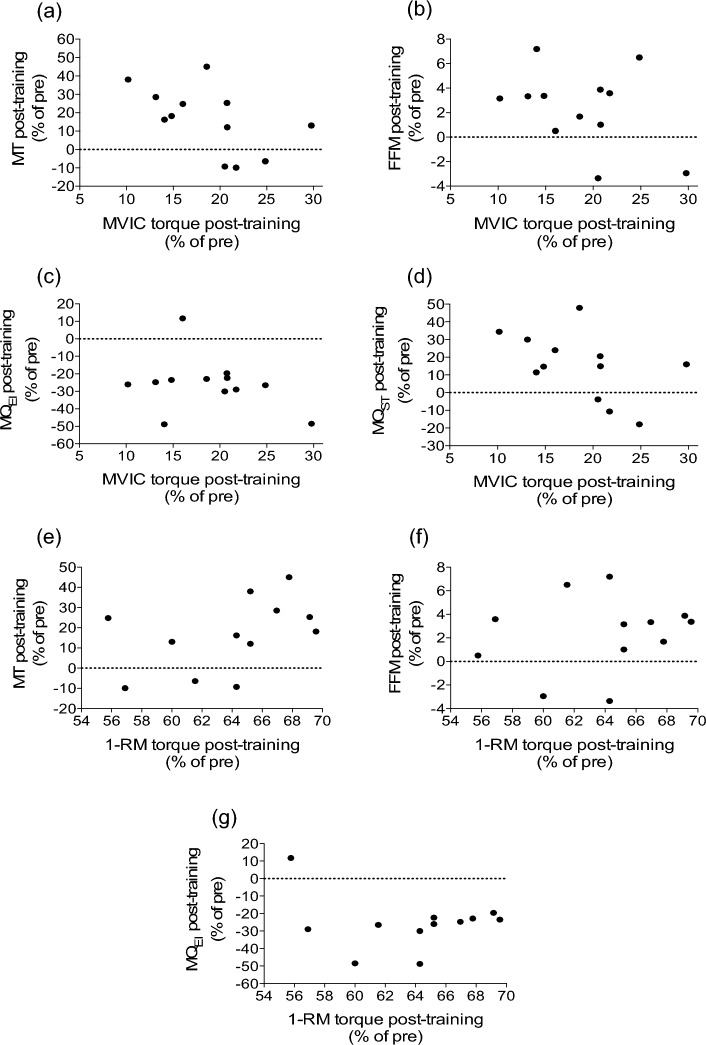
Significant linear mixed model associations illustrated by the magnitude of changes from before to after 12 weeks of training (post-training minus pre-training as % of pre-training value) in muscle thickness (MT, **a** and **e**), fat free mass (FFM, **b** and **f**), and muscle quality measured by echo intensity (MQ_EI_, **c** and **g**) and specific tension (MQ_ST_, **d**) in relation to the magnitude of changes from before to after changes in maximal voluntary isometric contraction (MVIC) torque and/or one-repetition maximum (1-RM)

Additional linear mixed model analyses revealed linear associations between the significant changes in knee extensor strength (1-RM and/or MVIC torque) and the significant changes in neuromuscular (MT, MQ_EI_ and MQ_ST_) and body composition (FFM) variables from pre- to post-training. These linear associations are illustrated in Fig. 5 by demonstrating the magnitude of changes between the associated variables from before to after 12 weeks of training (post-training minus pre-training as % of pre-training value). More specifically, parameter estimate analyses of the models showed that for every one unit change in MVIC torque (i.e., 1 N m), MT significantly increased by 1.78 mm [95% CI: 0.59–2.96; *t*(20) = 3.12, *p* = 0.005] (Fig. 5a), FFM increased by 0.23 kg [95% CI: 0.005–0.40; *t*(12) = 2.84, *p* = 0.02] (Fig. 5b), MQ_EI_ decreased by 1.77 A.U. [95% CI: −3.38 to −0.15; *t*(18) = −2.29, *p* = 0.03] (Fig. 5c) and MQ_ST_ increased by 0.66 N m/kg [95% CI: 0.05–0.08; *t*(15) = 9.90, *p* < 0.01] (Fig. 5d). In addition, for every one unit change in 1-RM (i.e., 1 kg), MT significantly increased by 0.14 mm [95% CI: 0.001–0.29; *t*(12) = 2.19, *p* = 0.04)] (Fig. 5e), FFM increased by 0.02 kg [95% CI: 0.001–0.02; *t*(13) = 3.17, *p* < 0.01] (Fig. 5f) and MQ_EI_ significantly decreased by 1.97 A.U. [95% CI: −3.21 to −0.73; *t*(14) = −3.42, *p* = 0.004)] (Fig. 5g). However, no significant linear association was observed between MQ_ST_ and 1-RM [95% CI: −1.35–1.85; *t*(27) = 0.32, *p* = 0.74].

## Discussion

We hypothesized in the present study that the 12-week structured RT would increase neuromuscular function and body composition of sedentary pubertal children. In line with the hypothesis, knee extension 1-RM and MVIC torque, total body FFM, quadriceps MT and MQ (using two commonly used methods for this assessment) increased from pre- to post-training. Additionally, linear mixed models showed significant associations between the changes in 1-RM and/or MVIC torque and the changes in quadriceps MT, MQ_EI_, MQ_ST_, and total body FFM following RT (Fig. 5). However, RTD, EMG activity, and other DXA-derived whole body or thigh composition variables did not change following the 12-week RT program. Therefore, the 12-week RT seemed to be effective for reducing the risk of pediatric dynapenia.

It should be noted that the participants of the present study had never performed any structural resistance exercise training program, and their baseline measures indicated that they were considered to be at risk of pediatric dynapenia (see Table 1). It has been reported that sedentary children and adolescents may have poor exercise compliance, especially when it involves prolonged periods of continuous exercise (Myer et al. 2015). However, all participants of the present study had a great adherence to the training program and did not miss any session. The reason for this may be due to the friendly and enjoyable atmosphere in the RT program, that was safe, well structured, and instructed by experienced instructors. Overall, our results were aligned with previous studies that recommend structured RT interventions for children to increase muscle strength, which is fundamental to develop physical capacities during childhood development (Faigenbaum et al. 2018a, 2019, 2023; Faigenbaum and MacDonald 2017). In fact, previous studies (Faigenbaum et al. 2023; Lesinski et al. 2020) have suggested that RT has the potential to enhance physical fitness in children beyond a level explained exclusively by growth and/or maturation. Thus, the integration of well-designed RT programs in school and community programs that target strength deficits and build strength reserves in youth have critical importance to improve physical fitness and prevent dynapenia in children (Faigenbaum et al. 2019, 2023). Clinicians and health care providers also have an important role in identifying physically inactive youth and referring them to exercise professionals who can design appropriate exercises programs targeted to improve muscular strength/power and neuromuscular deficiencies (Faigenbaum et al. 2018b).

Previous studies have recommended that structured RT interventions should be prescribed to prevent and/or treat pediatric dynapenia (Faigenbaum et al. 2018a, 2019; Faigenbaum and MacDonald 2017). However, to the best of our knowledge no previous experimental study has systematically examined effects of RT programs on children who had low muscle strength levels. In the present study, knee extensor MVIC torque was increased ~ 16% and leg extension 1-RM was increased ~ 64% following the 12-week (2 times/week) RT (Fig. 2a, b). It is difficult to compare our findings with previous RT studies in youth, because of the differences in the maturity level, training methods and outcome measures. However, the muscle strength improvements found in the present study were partly aligned with the results from Faigenbaum et al. (1999) who reported that ~ 5–12 year-old children had 31–42% greater leg extension 1-RM after performing twice-weekly RT sessions involving low or high volumes of training for 8 weeks. Similarly, high-intensity and circuit RT programs have been shown to be effective for increasing isokinetic knee extension peak torque by ~ 15% after 10 weeks (2 sessions/week) (Granacher et al. 2011), and by 21% after 20 weeks (3 sessions/week) in children (Ramsay et al. 1990). However, the lack of changes in RTD after the RT program of the present study seems to indicate that training programs that integrate explosive strength components such as speed, power and agility (e.g., fundamental integrative training) (Bukowsky et al. 2014) might be more effective to modify this variable in youth. Further research is necessary to confirm this assumption.

It is well known that pubertal children show increases in muscle tissue following RT, probably due to increases in the levels of circulating hormones and growth factors (Kraemer et al. 1989). As shown in Fig. 3a, quadriceps MT increased ~ 19% after 12 weeks of training, and based upon the individual data, all participants displayed hypertrophic responses. The results of the present study are consistent with those of a previous study (McKinlay et al. 2018) showing increases in vastus lateralis MT of 6.7–8.1% in young soccer players (11–13 years) who undertook 8 weeks of free-weight RT or plyometric training (2 sessions/week). In contrast, our results are not consistent with those of another study reporting no significant changes in quadriceps cross sectional area in 8-year-old children following 20 weeks of RT (Granacher et al. 2011). Taken together, these findings seem to support the contention that RT is more likely to modify muscle size during or after puberty compared to before puberty (Malina 2006).

The results of the present study also revealed that echo-intensity (i.e., MQ_EI_) decreased by ~ 26% and MQ_ST_ increased by ~ 15% from pre- to post-training, demonstrating an overall improvement in the quality of knee extensor muscles following RT (Fig. 3b, c). To the best of our knowledge, only one previous study (Mota et al. 2017) examined changes in MQ of healthy children, and reported that MQ_EI_ of the VL and RF did not change after 16 weeks of strength and conditioning program, while small increases in MQ_ST_ (i.e., knee extension peak torque divided by DXA-derived thigh lean mass) were found. However, in the present study we found for the first time that MQ_EI_ and MQ_ST_ improved in children after RT (Fig. 3). Physiological mechanisms underpinning this improvement remain to be elucidated. It may be that an increase in the contractile proteins with a concomitant reduction in intramuscular fat infiltration and/or increased ability to generate knee extensor MVIC torque relative to the “quadriceps lean mass” (N m kg^–1^) (Radaelli et al. 2013; Goodpaster et al. 2006; Mota et al. 2017) contributed to the improved MQ. A previous study (Weiss et al. 2005) found that the accumulation of intramyocellular lipid in the skeletal muscle was associated with development of muscle insulin resistance in obese pubertal children. Thus, it may be that monitoring and improving MQ_EI_ and MQ_ST_ by structured RT programs can be important for prevention or reversal of lipid deposits at cellular and molecular levels to avoid associated health complications. Nonetheless, these findings should be interpreted with caution due to the different approaches that are commonly used to assess MQ_EI_ and MQ_ST_, and the multiple methodological factors that can affect the interpretation of changes in EI (Pinto et al. 2022) and specific tension (Mota et al. 2017) in different muscles from before to after training.

Although not thoroughly investigated, previous studies have reported that RT improves body composition measured by DXA in children (dos Cunha et al. 2015; McGuigan et al. 2009). dos Cunha et al. (2015) showed that children (~ 10 years) that enrolled in a RT program for 12 weeks (3 sessions/week) had similar increases in FFM to a control group that performed no training. The authors suggested that these changes were due to biological maturation. However, the control group increased total fat mass by 4.6%, whereas no changes in fat mass were observed in the training group. In contrast, McGuigan et al. (2009) found that 8 weeks of RT in children that had the same age of the aforementioned study resulted in 2.6% decreases in percent body fat and 5.3% increases in FFM. Nonetheless, none of these studies showed changes in bone and/or other body composition variables. The present study found an increase in FFM by 2.3% after the RT program, but no significant changes in other DXA-derived body or thigh-specific bone composition variables (Fig. 4, Table 3). It is possible that a greater training volume is required to elicit such changes. Specific nutritional programs during RT might also be important when targeting changes in body and bone composition. In fact, Volek et al. (2003) reported that a 12-week RT program (3 times/week) combined with high calcium intake (i.e., 3 servings/day of 1% fluid milk) increased total body FFM by 5.1%, BMC by 3.6%, BMD by 1.7%, and reduced fat mass by 9.3% in adolescent boys.

The linear mixed model associations illustrated by the magnitude of changes from before to after 12 weeks of RT (Fig. 5) mostly showed that the improvements in strength (1-RM and/or MVIC torque) were linearly associated with MT, MQ_EI_, MQ_ST_ and FFM changes. Importantly, the baseline values of knee extensor MVIC/BM in the present study (boys: 2.68 ± 0.48 N m kg^–1^; girls: 2.34 ± 0.48 N m kg^–1^) were at a similar level or lower to those reported by most previous studies in the last two decades in severely obese untrained male and female adolescents, and lower than those previously reported in physically active nonobese adolescents from a similar age range (12–15 years) (Table 1). The only exception to this is the recent study from Gillen et al. (2020) in which physically active boys had very low MVIC/BM (~ 2.2 N m kg^–1^) compared to our sample and to the studies published in previous years. The baseline 1-RM (kg)/BM (kg) of the present study participants (boys: 0.62 ± 0.13; girls: 0.46 ± 0.06) was also lower when compared with physically active male adolescents reported by a previous study (Pullinen et al. 2011). Although the present study included a mixed sample of boys and girls and comparisons between studies with different methodologies are often difficult, the improvements in knee extension strength (MVIC/BM: ~ 15%, and 1-RM/BM: ~ 64%) in relation with some neuromuscular and body composition components following the 12-week RT program suggest that in general the children in our study could be at greater risk of pediatric dynapenia without engaging in the RT program. It is also important to note that for boys the post-RT MVIC/BM (3.58 ± 0.59 N m kg^–1^) and 1-RM/BM (1.84 ± 0.39 kg kg^−1^) reached levels close to those reported for physically active youth from similar age ranges in previous studies (Table 1), although these levels were much lower for girls (2.40 ± 0.58 N m kg^–1^ and 1.14 ± 0.27 kg kg^−1^). No sex comparisons were made in the present study because of the relatively small sample size of boys and girls. Thus, the comparison of neuromuscular adaptations to RT across sex in sedentary children may be of interest in future studies to explore whether different training strategies for boys and girls are necessary to offset pediatric dynapenia.

The magnitude of the changes in some of the outcome measures following the 12-week RT found in the present study was also similar to that reported by previous training studies in untrained older adults rather than young adults. For instance, Alegre et al. (2006) found that ~ 22 year-old healthy young adults (untrained in RT) increased squat 1-RM by 8.2% and VL MT by 6.9% following 13 weeks (3 sessions/week) of dynamic RT. Other studies in untrained adults from similar age ranges also showed that quadriceps cross sectional area (~ 8.8%) and knee extension MVIC (~ 13.5%) increased following 14 weeks of heavy-RT (Aagaard et al. 2001), but that no changes in MQ_EI_ occurred following short-term maximal isokinetic strength training (Ruas et al. 2018). In contrast, previous studies in untrained older men and/or women (60–74 years) have shown that leg extension 1-RM increases ~ 38–44% following 12-week RT programs (2 or 3 times/week) (Radaelli et al. 2013; Correa et al. 2016; Kalapotharakos et al. 2005). Other studies have also shown that the same time period/frequency of RT to the present study increased leg press MVIC (~ 14–21%) (Radaelli et al. 2013; Caserotti et al. 2008), quadriceps MT (14%) and MQ_ST_ (~ 20%), and decreased (i.e., indicating improvement) MQ_EI_ (~ 21%) (Radaelli et al. 2013). The similar magnitude of improvements in many neuromuscular variables between the children from the current study and older adults from previous studies following RT highlights the critical importance of increasing muscle strength at these two distinct life stages.

In fact, Faigenbaum et al. (2018a, 2019), Faigenbaum and MacDonald 2017) suggested that both older adults and youth share in common risk factors related to sedentarism and muscle weakness, and that both populations can be just as vulnerable to the inevitable consequences of neuromuscular dysfunction and dynapenia. Thus, it is recommended that both children and older adults should engage in regular RT programs (Faigenbaum et al. 2023; Fragala et al. 2019). In particular, RT can improve musculoskeletal, mental and cardiometabolic health in youth, which in turn may lead to improvements in muscular fitness and reduce risks of health complications related to muscle disuse in this population (Faigenbaum et al. 2023). Furthermore, well-designed RT programs can improve neuromuscular functioning, muscular strength and power in older adults, which can serve to counteract age-related declines in muscle mass, bone density, functional capacity, muscular function, cardiorespiratory fitness and reduce the risk of chronic health conditions, falls and fractures (Fragala et al. 2019).

A closer look at the data points revealed that the participants from the present study who had larger improvements in MT, FFM and MQ_EI_ and MQ_ST_ were those who had ~ 15–25% increases in MVIC torque, while the individuals who had the largest improvements in MVIC torque (~ 30%) did not necessarily show large improvements in MT, FFM, MQ_EI_ and MQ_ST_ variables (Fig. 5a–d). Additionally, the individuals who showed the greatest improvements in 1-RM (~ 70%) did not show large improvements in MT, FFM and MQ_EI_. This might suggest that pubertal children do not necessarily need to reach their maximal strength production capacity after RT programs to elicit optimal muscle size, lean mass and muscle quality.

It has been previously suggested that children are not capable of fully activating their motor units to the same extent as adults, in particular due to an inability to recruit higher threshold motor units (Dotan et al. 2012). However, it was not possible to detect differences in motor unit types with the measures examined in the present study, and the lack of changes in EMG activity (Fig. 2d) confirmed that RT-derived muscle strength improvements in children may have been more reflective from peripheral than central neuromuscular factors. Previous cross-sectional studies using nerve stimulation measures (i.e., twitch interpolation technique) have shown that the level of voluntary activation (i.e., which indicates differential recruitment of motor units) is lower in children (6–8 years old) than adults, but that these differences become less evident when children are older than 10 years. In addition, greater individual variability occurs in these measures due to differential maturity status (Ratel et al. 2015; Piponnier et al. 1985; Chalchat et al. 2019). Ramsay et al. (1990) used nerve stimulation measures to examine the mechanisms underlying strength gains in prepubertal children who undertook RT for 20 weeks (3 times/week). They found that children improved upper and lower limb dynamic and isokinetic muscle strength after training, but no significant changes in levels of voluntary activation were observed. Therefore, follow-up training studies including twitch interpolation technique combined with other neurophysiological measurements in youth are necessary to elucidate the mechanisms underpinning the strength gains found in children following RT. These measures will also allow us to improve our understanding on the reduced strength relative to neuromuscular and body composition adaptations after RT that occurred in some individuals as demonstrated in Fig. 5.

Several limitations in the present study should be mentioned. First, no neuromuscular or body composition measurements were included at other time points of the 12-week training period. The inclusion of additional time points might be important to further monitor the time course of changes leading to the body composition and neuromuscular adaptations in children over (and after the end of) the training intervention. Future research may also be necessary to further examine the effects of structured RT with longer durations (> 12 weeks) and/or that adopt different training strategies (e.g., fundamental integrative training) to enhance other fundamental physical variables in children such as power, speed and agility. Second, we did not include a control group or other groups of children with different age ranges/maturity levels for comparison with the experimental group in the present study due to difficulties in the recruitment process of children of equivalent age and Tanner stage. The inclusion of a control group would be important to completely isolate the effects of the RT program from any other influence on the participant’s development (e.g., maturation) during the course of the study. Although caution was taken to compare pre- to post-training changes in the tested variables by the use of appropriate statistical models that reduce inter-individual variability and eliminate confounding variables, it is still possible that the interpretation of our results could be affected by individual variability of children in response to RT and/or the specific maturation status of our sample. However, it is likely that maturation had little effect on the results of the present study since an additional analysis revealed that (i) no significant changes in thigh-specific bone height measures (i.e., the distance from the greater trochanter to the medial line of the patella; Table 3), and (ii) no significant linear associations were obtained between thigh-specific bone height measures and changes in knee extensor strength (1-RM and MVIC torque) from pre- to post-training (all *p* > 0.059). Third, it seems possible that responses to RT are different between boys and girls, thus larger sample size for each sex is necessary in the future studies. Furthermore, although most of the measurements included in the present study have been shown to be highly reliable in children by previous studies using similar settings as the ones from the present study (dos Cunha et al. 2015; Stock et al. 2017; Margulies et al. 2005), it would be important to investigate the relative and absolute reliability of the outcome measures particularly in children with dynapenia. Finally, the present study included many measurements contributing to the understanding of the mechanisms underpinning strength adaptations following RT in youth. Nonetheless, additional neurophysiological assessments at the supraspinal and spinal levels could be required to further explore strength improvements relative to neuromuscular and body composition adaptations.

In conclusion, the findings of the present study showed that the 12-week RT was effective for increasing knee extension 1-RM and MVIC torque, which were associated with improvements in total body FFM, and quadriceps MT, MQ_ST_ and MQ_EI_ in untrained pubertal children. In particular, the large improvements in MQ (using two commonly used assessments) seem to indicate that children at risk of dynapenia may reduce intramuscular lipid infiltration in the skeletal muscle and/or improve their ability to generate force relative to lean mass after engaging in a RT program, which could potentially decrease risks of health complications and disorders associated to this condition. Furthermore, it appears that children do not need to reach their maximal strength production capacity at the end of the training period to elicit maximal adaptations in muscle size and quality, and improve their total body FFM. Therefore, the associated improvements in overall strength, neuromuscular and body composition variables found in the present study indicate that the RT seems to reduce the risk of pediatric dynapenia in pubertal children.

## Data Availability

All data generated or analyzed during this study are included in the article.

## Abbreviations

RT: Resistance training
1-RM: One-repetition maximum
MVIC: Maximal voluntary isometric contraction
RTD: Rate of torque development
EMG: Electromyographic
MT: Muscle thickness
EI: Echo-intensity
MQ: Muscle quality
MQ_EI_: MQ assessed by echo intensity
MQ_ST_: MQ assessed by specific tension of MVIC torque to thigh fat-free mass
BMI: Body mass index
FFM: Fat-free mass
BMD: Bone mineral density
BMC: Bone mineral content
VL: Vastus lateralis
VM: Vastus medialis
RF: Rectus femoris
VI: Vastus intermedius

## References

[CR1] Aagaard P, Andersen JL, Dyhre-Poulsen P (2001). A mechanism for increased contractile strength of human pennate muscle in response to strength training: changes in muscle architecture. J Physiol.

[CR2] Abdelmoula A, Martin V, Bouchant A (2012). Knee extension strength in obese and nonobese male adolescents. Appl Physiol Nutr Metab.

[CR3] Alegre LM, Jiménez F, Gonzalo-Orden JM, Martín-Acero R, Aguado X (2006). Effects of dynamic resistance training on fascicle length and isometric strength. J Sports Sci.

[CR4] Aubert S, Barnes JD, Abdeta C (2018). Global matrix 3.0 physical activity report card grades for children and youth: results and analysis from 49 countries. J Phys Act Health.

[CR5] Bukowsky M, Faigenbaum AD, Myer GD (2014). FUNdamental integrative training (FIT) for physical education. J Phys Educ Recreat Dance.

[CR6] Caserotti P, Aagaard P, Larsen JB, Puggaard L (2008). Explosive heavy-resistance training in old and very old adults: changes in rapid muscle force, strength and power. Scand J Med Sci Sports.

[CR7] Chalchat E, Piponnier E, Bontemps B (2019). Characteristics of motor unit recruitment in boys and men at maximal and submaximal force levels. Exp Brain Res.

[CR8] Cohen DD, Voss C, Taylor MJ, Delextrat A, Ogunleye AA, Sandercock GR (2011). Ten-year secular changes in muscular fitness in English children. Acta Paediatr.

[CR9] Colley RC, Garriguet D, Janssen I, Craig CL, Clarke J, Tremblay MS (2011). Physical activity of Canadian adults: accelerometer results from the 2007 to 2009 Canadian health measures survey. Health Rep.

[CR10] Correa CS, Cunha G, Marques N, Oliveira-Reischak Ã, Pinto R (2016). Effects of strength training, detraining and retraining in muscle strength, hypertrophy and functional tasks in older female adults. Clin Physiol Funct Imaging.

[CR11] dos Cunha SG, Sant'anna MM, Cadore EL (2015). Physiological adaptations to resistance training in prepubertal boys. Res Q Exerc Sport.

[CR12] Dotan R, Mitchell C, Cohen R, Klentrou P, Gabriel D, Falk B (2012). Child–adult differences in muscle activation - a review. Pediatr Exerc Sci.

[CR13] Faigenbaum AD, MacDonald JP (2017). Dynapenia: it's not just for grown-ups anymore. Acta Paediatr.

[CR14] Faigenbaum AD, Westcott WL, Loud RL, Long C (1999). The effects of different resistance training protocols on muscular strength and endurance development in children. Pediatrics.

[CR15] Faigenbaum AD, Rebullido TR, MacDonald JP (2018). Pediatric inactivity triad: a risky PIT. Curr Sports Med Rep.

[CR16] Faigenbaum AD, Rial Rebullido T, MacDonald JP (2018). The unsolved problem of paediatric physical inactivity: it's time for a new perspective. Acta Paediatr.

[CR17] Faigenbaum AD, Rebullido TR, Peña J, Chulvi-Medrano I (2019). Resistance exercise for the prevention and treatment of pediatric dynapenia. J Sci Sports Exer.

[CR18] Faigenbaum AD, Ratamess NA, Kang J, Bush JA, Rial RT (2023). May the force be with youth: foundational strength for lifelong development. Curr Sports Med Rep.

[CR19] Fragala MS, Cadore EL, Dorgo S (2019). Resistance training for older adults: position statement from the national strength and conditioning association. J Strength Cond Res.

[CR20] Fraser BJ, Blizzard L, Buscot MJ (2021). The association between grip strength measured in childhood, young- and mid-adulthood and prediabetes or type 2 diabetes in mid-adulthood. Sports Med.

[CR21] García-Hermoso A, Ramírez-Campillo R, Izquierdo M (2019). Is muscular fitness associated with future health benefits in children and adolescents? A systematic review and meta-analysis of longitudinal studies. Sports Med.

[CR22] Garcia-Vicencio S, Martin V, Kluka V (2015). Obesity-related differences in neuromuscular fatigue in adolescent girls. Eur J Appl Physiol.

[CR23] Gillen ZM, Shoemaker ME, Bohannon NA, Gibson SM, Cramer JT (2020). Comparing the torque- and power-velocity relationships between children and adolescents during isokinetic leg extension muscle actions. Hum Mov Sci.

[CR24] Goodpaster BH, Park SW, Harris TB (2006). The loss of skeletal muscle strength, mass, and quality in older adults: the health, aging and body composition study. J Gerontol A Biol Sci Med Sci.

[CR25] Granacher U, Goesele A, Roggo K (2011). Effects and mechanisms of strength training in children. Int J Sports Med.

[CR26] Henriksson H, Henriksson P, Tynelius P, Ortega FB (2019). Muscular weakness in adolescence is associated with disability 30 years later: a population-based cohort study of 1.2 million men. Br J Sports Med..

[CR27] Hermens HJ, Freriks B, Merletti R (1999). European recommendations for surface electromyography. Roessingh Res Dev.

[CR28] Janssen X, Mann KD, Basterfield L (2016). Development of sedentary behavior across childhood and adolescence: longitudinal analysis of the gateshead millennium study. Int J Behav Nutr Phys Act.

[CR29] Kalapotharakos VI, Michalopoulos M, Tokmakidis SP, Godolias G, Gourgoulis V (2005). Effects of a heavy and a moderate resistance training on functional performance in older adults. J Strength Cond Res.

[CR30] Katzmarzyk PT, Denstel KD, Beals K (2018). Results from the United States 2018 report card on physical activity for children and youth. J Phys Act Health.

[CR31] Kraemer WJ, Fry AC, Frykman PN, Conroy B, Hoffman J (1989). Resistance training and youth. Pediatr Exerc Sci.

[CR32] Laurson KR, Saint-Maurice PF, Welk GJ, Eisenmann JC (2017). R Reference curves for field tests of musculoskeletal fitness in U.S. children and adolescents: the 2012 NHANES national youth fitness survey. J Strength Cond Res.

[CR33] Lesinski M, Herz M, Schmelcher A, Granacher U (2020). Effects of resistance training on physical fitness in healthy children and adolescents: an umbrella review. Sports Med.

[CR34] Maffiuletti NA, Jubeau M, Agosti F, De Col A, Sartorio A (2008). Quadriceps muscle function characteristics in severely obese and nonobese adolescents. Eur J Appl Physiol.

[CR35] Magezi DA (2015). Linear mixed-effects models for within-participant psychology experiments: an introductory tutorial and free, graphical user interface (LMMgui). Front Psychol.

[CR36] Malina RM (2006). Weight training in youth-growth, maturation, and safety: an evidence-based review. Clin J Sport Med.

[CR37] Margulies L, Horlick M, Thornton JC, Wang J, Ioannidou E, Heymsfield SB (2005). Reproducibility of pediatric whole body bone and body composition measures by dual-energy X-ray absorptiometry using the GE lunar prodigy. J Clin Densitom.

[CR38] Matthews CE, Chen KY, Freedson PS (2008). Amount of time spent in sedentary behaviors in the United States, 2003–2004. Am J Epidemiol.

[CR39] McGuigan MR, Tatasciore M, Newton RU, Pettigrew S (2009). Eight weeks of resistance training can significantly alter body composition in children who are overweight or obese. J Strength Cond Res.

[CR40] McKinlay BJ, Wallace P, Dotan R (2018). Effects of plyometric and resistance training on muscle strength, explosiveness, and neuromuscular function in young adolescent soccer players. J Strength Cond Res.

[CR41] Mota JA, Stock MS, Thompson BJ (2017). Vastus lateralis and rectus femoris echo intensity fail to reflect knee extensor specific tension in middle-school boys. Physiol Meas.

[CR42] Moura BM, Ruas CV, Diefenthaeler F (2021). Influence of muscle strength gains on functional capacity improvements following resistance training in older adults: a linear mixed model approach. Phys Occup Ther Geriatr.

[CR43] Moyer MW (2022). The COVID generation: how is the pandemic affecting kids' brains?. Nature.

[CR44] Myer GD, Faigenbaum AD, Edwards NM, Clark JF, Best TM, Sallis RE (2015). Sixty minutes of what? A developing brain perspective for activating children with an integrative exercise approach. Br J Sports Med.

[CR45] Pinto MD, Pinto RS, Nosaka K, Blazevich AJ (2022). Do intramuscular temperature and fascicle angle affect ultrasound echo intensity values?. Med Sci Sports Exerc..

[CR46] Piponnier E, Martin V, Bontemps B (1985). Child-adult differences in neuromuscular fatigue are muscle-dependent. J Appl Physiol.

[CR47] Pullinen T, Mero A, Huttunen P, Pakarinen A, Komi PV (2011). Resistance exercise-induced hormonal response under the influence of delayed onset muscle soreness in men and boys. Scand J Med Sci Sports.

[CR48] Radaelli R, Botton CE, Wilhelm EN (2013). Low- and high-volume strength training induces similar neuromuscular improvements in muscle quality in elderly women. Exp Gerontol.

[CR49] Ramsay JA, Blimkie CJ, Smith K, Garner S, MacDougall JD, Sale DG (1990). Strength training effects in prepubescent boys. Med Sci Sports Exerc.

[CR50] Ratel S, Kluka V, Vicencio SG (2015). Insights into the mechanisms of neuromuscular fatigue in boys and men. Med Sci Sports Exerc.

[CR51] Ruas CV, Pinto RS, Lima CD, Costa PB, Brown LE (2017). Test–retest reliability of muscle thickness, echo-intensity and cross sectional area of quadriceps and hamstrings muscle groups using b-mode ultrasound. Int J Kines Sport Sci.

[CR52] Ruas CV, Brown LE, Lima CD, Gregory Haff G, Pinto RS (2018). Different muscle action training protocols on quadriceps-hamstrings neuromuscular adaptations. Int J Sports Med.

[CR53] Ruas CV, Pinto RS, Haff GG, Lima CD, Brown LE (2019). Effects of different combinations of concentric and eccentric resistance training programs on traditional and alternative hamstrings-to-quadriceps ratios. Sports (Basel).

[CR54] Shuffrey LC, Firestein MR, Kyle MH (2022). Association of birth during the COVID-19 pandemic with neurodevelopmental status at 6 months in infants with and without in utero exposure to maternal SARS-CoV-2 infection. JAMA pediatr..

[CR55] Stock MS, Mota JA, Hernandez JM, Thompson BJ (2017). Echo intensity and muscle thickness as predictors of athleticism and isometric strength in middle-school boys. Muscle Nerve.

[CR56] Sum KK, Cai S, Law E (2022). COVID-19-related life experiences, outdoor play, and long-term adiposity changes among preschool- and school-aged children in singapore 1 year after lockdown. JAMA Pediatr.

[CR57] Tanner JM (1962). Growth at adolescence.

[CR58] Timpka S, Petersson IF, Zhou C, Englund M (2014). Muscle strength in adolescent men and risk of cardiovascular disease events and mortality in middle age: a prospective cohort study. BMC Med.

[CR59] Volek JS, Gómez AL, Scheett TP (2003). Increasing fluid milk favorably affects bone mineral density responses to resistance training in adolescent boys. J Am Diet Assoc.

[CR60] Weiss R, Taksali SE, Dufour S (2005). The “obese insulin-sensitive” adolescent: importance of adiponectin and lipid partitioning. J Clin Endocrinol Metab.

